# Mechanochemical ablation versus cyanoacrylate adhesive for the treatment of varicose veins: study protocol for a randomised controlled trial

**DOI:** 10.1186/s13063-018-2807-0

**Published:** 2018-08-07

**Authors:** Amjad Belramman, Roshan Bootun, Tjun Yip Tang, Tristan R. A. Lane, Alun H. Davies

**Affiliations:** 1 Section of Vascular Surgery, Department of Surgery and Cancer, Imperial College London, 4N12A, Charing Cross Hospital, Fulham Palace Road, London, W6 8RF UK; 2 East of England Deanery, London, UK; 3 Singapore General Hospital, London, Singapore; 4 Sengkang General Hospital, Singapore, Republic of Singapore; 5 London Deanery, London, UK; 6 Imperial College Healthcare NHS Trust, London, UK

**Keywords:** Endovenous ablation, Varicose veins, Venous disease, Mechanochemical ablation, Cyanoacrylate adhesive

## Abstract

**Background:**

Thermal ablation techniques have become the first-line treatment of truncal veins in the management of chronic venous disease (CVD). Despite excellent outcomes, these methods are often associated with pain; generally due to their use of heat and the necessity of fluid infiltration around the vein. More recently, novel non-thermal techniques, such as mechanochemical ablation (MOCA) and cyanoacrylate adhesive (CAE) have been developed to overcome these unwelcome effects. So far, the novel techniques have been found to have similar efficacy to thermal methods, yet no direct comparisons between the non-thermal treatment techniques have been conducted to date, giving rise to this study.

**Methods/design:**

This is a prospective, multicentre, randomised clinical trial, recruiting patients with truncal saphenous incompetence. Patients will be randomised to undergo either MOCA or CAE truncal ablation, followed by treatment of any varicosities. All patients will be required to wear compression stockings for 4 days post intervention. The primary outcome measure is the pain score immediately following completion of truncal ablation, measured by a 100-mm Visual Analogue Scale (VAS). The secondary outcomes are entire treatment pain scores, clinical scores, quality of life scores, occlusion rates, time to return to usual activities/work at 2 weeks, 3, 6 and 12 months. Re-intervention rate will be considered from the third month. Cost-effectiveness will be assessed for each intervention at 12 months. The study is powered to detect a mean 10-mm difference in maximum pain score. Allowing for loss to follow-up, the total target recruitment is 180 patients.

**Discussion:**

The study will be the first study to compare MOCA against CAE and is designed to determine which method causes less pain. Completion of this study is expected to be the end of 2019.

**Trial registration:**

ClinicalTrials.gov, ID: NCT03392753. Registered on 17 November 2017.

**Electronic supplementary material:**

The online version of this article (10.1186/s13063-018-2807-0) contains supplementary material, which is available to authorized users.

## Background

Varicose veins are common and are known to affect approximately one third of the population [1]. Chronic venous disease (CVD) has been demonstrated to have a negative impact on the quality of life (QoL) of patients and treatment of varicose veins has been demonstrated to lead to improvement in the QoL of patients [2–4]. Over the past decade, new endovenous techniques have been introduced, and these are felt to be cost-effective, especially when performed in an outpatient or ‘office-based’ setting [5]. These endovenous ablation methods (almost exclusively radiofrequency ablation (RFA) or endovenous laser ablation (EVLA)) employ mainly thermal energy to treat varicose veins. The American Venous Forum (AVF) and the National Institute of Clinical Excellence (NICE) guidelines published in July 2013 recommended the use of endovenous thermal ablation techniques, namely radiofrequency ablation (RFA) or endovenous laser ablation (EVLA), as first-line treatment for truncal reflux [6–8]. Occlusion rates of greater than 90% have been demonstrated in studies looking at these two methods at up to 5 years of follow-up [9–14].

However, because these methods make use of thermal energy to denature the venous wall, they have the potential to cause pain, skin burns, skin pigmentation, nerve damage and even arteriovenous fistula formation [15, 16]. To minimise these possible complications and to allow effective treatment, tumescent anaesthesia must be infiltrated around the vein to be treated. This, in turn, can be a source of discomfort to patients.

More recently, newer non-thermal, non-tumescent ablation techniques (NTNTs) have been introduced in an attempt to reduce these complications. Mechanochemical ablation (MOCA) and cyanoacrylate adhesive injection (CAE) are two examples of these NTNTs [17, 18]. So far, pain has been shown to be less than [19], or comparable to [18], thermal methods, but also equivalent to them in terms of QoL improvement, time to return to normal activities and occlusion rates [18, 19]. This indicates that they may, one day, be considered favourable to endothermal ablation.

NICE has also recently produced interventional procedure guidance for the use of both MOCA [20] and CAE [21].

To date, there has not been a direct head-to-head comparison of these two non-thermal methods. We, propose to undertake a randomised controlled study comparing MOCA and CAE in the treatment of varicose veins.

### Objectives

The main aim of this study is to detect which method causes less pain. Secondary objectives are to assess the pain level over the ensuing few days, change in QoL, clinical severity score, degree of bruising and inflammation, time to return to normal activities/work, re-intervention rates as well as the cost-effectiveness of each method.

### Trial design

**Fig. 1:**
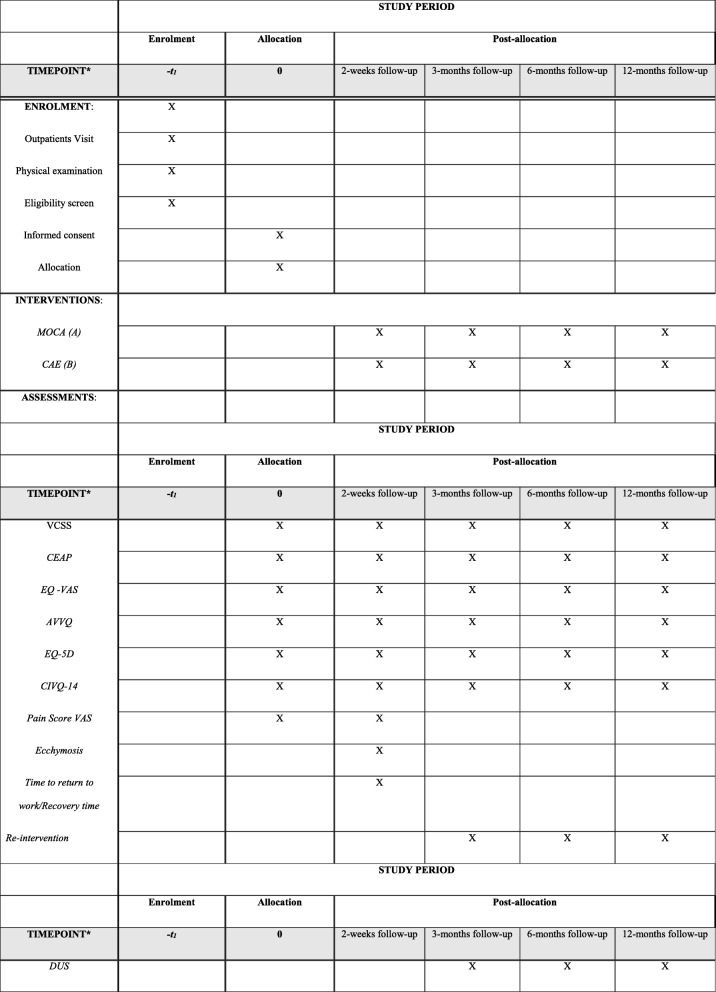
Schedule of enrolment, interventions and assessments according to the SPIRIT 2013 Statement: Defining Standard Protocol Items for Clinical Trials. *CEAP* Clinical Etiology Anatomy Pathophysiology, *VCSS* Venous Clinical Severity Score, *EQ-VAS* EuroQol’s Visual Analogue Scale, *AVVQ* Aberdeen Varicose Vein Questionnaire, *CIVQ-14* Chronic Venous Insufficiency Questionnaire, *EQ-5D* EuroQol’s 5-domain Utility Index, *Pain Score VAS* Visual Analogue Scale, *DUS* Duplex ultrasound, *MOCA* mechanochemical ablation, *CAE* cyanoacrylate adhesive

This is a prospective, multicentre, randomised clinical trial comparing MOCA to CAE in the treatment of truncal saphenous incompetence. It follows the Standard Protocol Items: Recommendations for Interventional Trials (SPIRIT) guidelines shown in the SPIRIT Checklist and Figure in Additional file 1 and Fig. 1, respectively. Patients are randomised into group A (MOCA) or group B (CAE). Only the endovenous ablation part of the procedure will be randomised, while the decision as to whether patients should receive treatment of their varicose tributaries will be at the discretion of the clinical team.

## Methods/design

### Study settings

This study is conducted at Charing Cross Hospital (Imperial College London, UK), Singapore General Hospital and Sengkang General Hospital (Singapore).

### Eligibility criteria

Patients who have primary great saphenous (GSV) or short saphenous vein (SSV) vein reflux > 0.5 s on Duplex ultrasound (DUS) scanning and who are aged over 18 years will be included. Exclusion criteria are: current deep vein thrombosis; recurrent varicose veins; arterial disease (ABPI < 0.8); venous diameter < 3 mm; patients who are unwilling to participate; inability or unwillingness to complete questionnaires; adverse reaction to sclerosant or cyanoacrylate or involvement in another venous trial in the past 6 months.

### Interventions

All interventions are performed under ultrasound guidance and local anaesthesia and are carried out by vascular surgeons who are experienced in both methods.

For both methods, the truncal vein (GSV or SSV) is cannulated under ultrasound guidance using the Seldinger technique after injection of local anaesthetic (1% lidocaine). The catheter-wire tip is placed 5 cm for the CAE group or 2 cm for the MOCA group distal to the sapheno-femoral junction (SFJ) or sapheno-popliteal junction (SPJ). Cannulation is obtained at the most distal point of venous reflux.

All patients are treated with the ClariVein® mechanochemical ablation device (Vascular Insights, Madison, CT, USA), or the VenaSeal™ Closure System (Medtronic, Minneapolis, MN, USA). The methods used for MOCA and for CAE are described in detail by van Eekeren et al. [22] and by Almeida et al. [23], respectively.

Following all procedures, an ultrasound scan is performed to assess both the treated truncal vein and the deep veins. Concomitant phlebectomy or foam sclerotherapy will then be carried out if deemed necessary and clinically indicated by the clinical team.

The duration of the procedure is defined as from the time of insertion of the cannula into the vein to the time of removal the catheter. The total volume of liquid sclerosant, the amount of proprietary cyanoacrylate glue and the length of the treated vein are recorded.

Re-intervention of the treated saphenous veins will not be considered until at least 3 months after the initial procedure.

### Primary outcome

The primary outcome measure is to record the pain score immediately following completion of the endovenous ablation using a 100-mm Visual Analogue Scale (VAS) [19, 22, 24].

### Secondary outcome

The secondary outcomes are to compare the two treatment groups with respect to:

- The pain score at the end of the procedure (including tributary treatment)
- QoL scores at baseline, 2 weeks, 3 months, 6 months and 12 months using the EuroQol 5-domain Utility Index (EQ-5D), the Aberdeen Varicose Vein Questionnaire (AVVQ) and the Chronic Venous Insufficiency Questionnaire (CIVIQ-14) scores
- Clinical change using the Venous Clinical Severity Score (VCSS) at baseline, 2 weeks, 3 months, 6 months and 12 months
- The pain score over the first 10 days, recorded as a number on a scale of 0–10 (0 means no pain, 10 means worst imaginable pain) [25]
- The degree of bruising at 2 weeks using an ecchymosis score with a 5-point scale [26]
- The time taken to return to work and normal activities
- Occlusion rates at 3 months, 6 months and 12 months
- Re-intervention rate at 12 months
- Comparison of the cost-effectiveness of each intervention at 12 months

### Sample size and study duration

The study is designed to assess for equivalence. Based on previous studies, 10 mm is a clinically appropriate minimum important difference [19, 22, 24]. We estimated the sample size needed to observe a mean difference of at least 10 mm (standard deviation: 20 mm) between the two interventions. With power set at 80% and 5% significance equivalence, we need to recruit 128 patients (64 patients per group). If a dropout rate from follow-up of 30% is estimated, the total number to recruit is 183. If at least three patients are recruited per week, 156 patients could potentially be randomised over 1 year, and 180 patients over 60 weeks (approximately 14 months). Thus, with 12-month follow-up the study will run for 26 months with a target recruitment of 180 patients.

### Recruitment

Patients referred for treatment of symptomatic varicose veins are recruited if they are found to have primary GSV or SSV incompetence on the DUS. Patients are identified in clinic and provided with further information regarding the study.

**Fig. 2:**
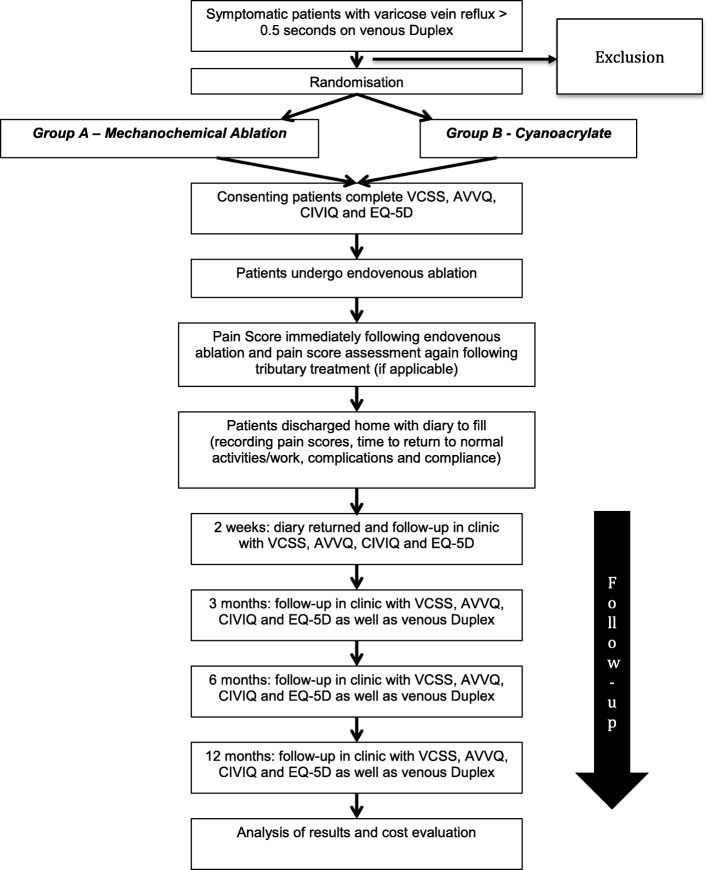
Trial flowchart

On the day of their procedures, patients are asked to sign a study consent form for inclusion in the study. They will then be allocated a study number. Patients are randomised to receive MOCA (group A) or CAE (group B) to treat their saphenous veins (Fig. 2).

At baseline, basic demographic data is collected from each patient. Patients are also be asked to provide their contact details to enable correspondence for follow-up appointments.

Patients are asked to complete QoL questionnaires using the EQ-5D, AVVQ and the CIVIQ-14, and have their clinical scores assessed using Clinical Etiological Anatomical and Pathophysiological (CEAP) classification and VCSS.

### Assessment of pain

Immediately following completion of the endovenous ablation, patients are requested to record their pain score using a 100-mm VAS. Once the pain score has been recorded, concomitant phlebectomy or foam sclerotherapy will then be carried out if deemed appropriate by the clinical team. At the end of this part of the procedure, the pain score is again assessed, using the VAS and number scale.

### Post procedure

Patients are allowed to leave hospital once they have spent a complication-free period in the department. On discharge, all patients are provided with compression stockings (class II 18–24 mmHg) to wear for 4 days. They are also being provided with a diary to record their post-procedural pain every day for the first 10 post-operative days using a validated VAS and to record when they return to their normal activities and are back to work. They are encouraged to mobilise as much as possible and to resume their normal activities when they feel capable of doing so.

### Follow-up treatment periods

Patients are requested to attend a follow-up for research purposes at 2 weeks, 3 months, 6 and 12 months (Fig. 1). The patient’s general practitioner will also be sent a letter to inform them of their patient’s participation in the study, with the consent of the patient.

### Follow-up at 2 weeks

At the 2-week follow-up, the diary containing details of the pain scores and how soon patients were able to return to normal activities/work are collected. In addition, patients are asked about, and examined for, any bruising (using an ecchymosis score with a 5-point scale) [26] or phlebitis that followed their procedure. They will undergo a clinical examination and the VCSS will be recorded and will be asked to fill in the EQ-5D, AVVQ and CIVIQ-14 scores. No decision regarding re-treatment will be taken at this point.

### Follow-up at 3 months, 6 months and 12 months

At the 3-month, 6-month and 12-month follow-ups, patients undergo further clinical examination, and their VCSS is recorded. They are asked to complete the EQ-5D, AVVQ and the CIVIQ-14 scores. They also undergo further venous DUS scanning to confirm occlusion of the treated vein.

From the third month following treatment, patients found to have recannulation of their treated truncal veins will be assessed to establish if they are symptomatic and require re-intervention. The method used for re-intervention will be dependent on the choice of the consultant in charge of the patient.

### Randomisation

On the day of treatment, written consent forms are obtained. Patients are then allocated randomly to one of the two intervention groups by equal randomisation using an online computerised randomisation system (SealedEnvelope, London, UK).

### Blinding

Blinding after randomisation to interventions is not possible from either the participant or the clinical team because of nature of the interventions.

Following the intervention, clinician review and DUS will be performed independently by assessors who are blinded to the group allocation.

### Data collection and confidentiality

All patient data will be anonymised and stored in a password-protected database under the guidelines of the Data Protection Act 1998. Patient records will be kept on paper in the form of the diary card questionnaires and clinical scoring sheets. These will be kept in a locked filing cabinet at Charing Cross Hospital for 10 years in accordance with Imperial College London’s policy.

The chief investigator will preserve the confidentiality of participants taking part in the study and is registered under the Data Protection Act.

Patient details will be anonymised as each participant will be allocated a study number. The allocated study number key code will be kept on a password-protected NHS computer at all sites.

Patient details, including contact information, will be recorded on the paper form. This paper form will be kept in a locked filing cabinet in a locked vascular research office, located in the Section of Vascular Surgery on Floor 4 North at Charing Cross Hospital (university office). The contact details will be discarded once patients have been advised of the findings of the study (within approximately 6–12 months following completion of the study).

All anonymised patient details with the allocated study number used as an identifier will be stored electronically on a password-protected access database on an Imperial College London university computer.

Data and study findings will be presented locally within the hospital, as well as at national and international peer-reviewed presentations.

All participants will be sent reminders by post or contacted over the telephone to attend the follow-up visits. In case if they fail to attend a scheduled visit, QoL questionnaires will be sent by post to be completed.

### Statistical analysis

Data will be analysed using SPSS version 24 (IBM, Armonk, NY, USA) STATA 15SE (StataCorp, College Station, TX, USA) or a similar statistical software. Data will be analysed on an intention-to-treat (ITT) basis. Visual testing and Shapiro-Wilk testing will be performed to assess the distribution of the data. For continuous data, if the data is normally distributed mean and standard deviation (SD) will be presented, whereas median and interquartile range (IQR) will be presented if it not normally distributed. For categorical data, frequencies and percentages will be presented. *T* tests may be conducted if the data is normally distributed, whereas the Mann Whitney *U* test may be more desirable if data is not normally distributed. A repeated measure analysis of variance (ANOVA) will be used to examine changes in scores from baseline during follow-up. All time-to-event outcomes will be assessed using Kaplan-Meier curves and log-rank tests for group comparison. Chi-squared tests will be performed to compare treatment group proportions. Missing data will be handled with multiple imputation methods.

### Cost-effectiveness analysis

Cost-effectiveness analysis for both interventions will be assessed using the cost of the equipment and the time required to perform the procedure, the cost of personnel involved, the cost of theatre usage, time to return to work and QoL gain following the procedure.

### Data monitoring, safety and quality control

An adverse event (AE) is an untoward medical occurrence in a patient or clinical study subject, which may or may not be caused by the investigational device. All such events, whether expected or not, should be recorded.

A serious adverse event (SAE) is an untoward and unexpected medical occurrence or effect that results in death or is life-threatening; specifically referring to an event in which the subject was at risk of death at the time of the event. It does not refer to an event which hypothetically might have caused death if it were more severe, requires hospitalisation or prolongation of existing inpatients’ hospitalisation, results in persistent or significant disability or incapacity, or results from a congenital anomaly or birth defect.

All AEs should be reported. Depending on the nature of the event the reporting procedures below should be followed. Any questions concerning AE reporting should be directed to the chief investigator in the first instance.

An SAE form should be completed and sent by fax or email to the chief investigator within 24 h. All SAEs should be reported to the Research Ethical Committee where, in the opinion of the chief investigator, the event was ‘related’ (i.e. resulted from the administration of any of the research procedures) and ‘unexpected’ (i.e. an event that is not listed in the protocol as an expected occurrence).

Reports of related and unexpected SAEs should be submitted within 15 days of the chief investigator becoming aware of the event, using the NRES SAE form for non-IMP studies.

Local investigators should report any SAEs as required by their Local Research Ethics Committee, sponsor and/or Research and Development Office.

In the event of any harm to participants in the trial, Imperial College holds public liability (‘negligent harm’) and clinical trial (‘non-negligent harm’) insurance policies which apply to this trial.

The study will be monitored and audited with Joint Research Compliance Office (JRCO) policy.

### Ethical approval and study registration

Ethical approval has been sought from a Regional Research Ethics Committee London (REC Ref: 17/LO/1457). The study has been registered at the ClinicalTrials.gov website (https://clinicaltrials.gov/show/NCT03392753). The study will be conducted in accordance with the recommendations for physicians involved in research on human subjects adopted by the 18th World Medical Assembly, Helsinki 1964 and later revisions.

## Discussion

Varicose veins are a very common condition associated with detrimental effects on the QoL of patients [2–4]. It has been proven that their management leads to improvement in QoL [2–4]. Recently, the newer techniques of MOCA and CAE have been introduced and do not require tumescent anaesthesia or the use of thermal energy. This has led to a reduction in patient discomfort, hematoma formation, and risk of a nerve injury when compared to thermal-based procedures.

The results, so far, of these new techniques have shown to be effective and safe [18, 19]; however, there is little data regarding their efficiency and durability.

Therefore, we have designed this multicentre randomised controlled trial to ascertain which one of these techniques causes less pain. To our knowledge, this is the first trial making the direct comparison between MOCA and CAE.

The aims of this current trial are firstly to compare the pain score between MOCA versus CAE and, secondly, to assess the pain level over the ensuing few days, change in the QoL, VCSS, degree of bruising and inflammation, time to return to normal activities/work, re-intervention rates and cost-effectiveness of each method.

The trial will be conducted across two very different healthcare systems in the UK and Singapore, broadening the population base, characteristics and application of findings.

### Trial status

At the time of submission, the study has commenced recruitment of participants in November 2017. The three collaborating centres are Charing Cross Hospital (Imperial College London, UK), Singapore General Hospital and Sengkang General Hospital (Singapore).

## Additional file

Additional file 1: Standard Protocol Items: Recommendations for Interventional Trials (SPIRIT) 2013 Checklist: recommended items to address in a clinical trial protocol and related documents. (DOC 123 kb)

## Abbreviations

ABPI: Ankle Brachial Pressure Index
AE: Adverse events
AVF: American Venous Forum
AVVQ: Aberdeen Varicose Vein Questionnaire
CAE: Cyanoacrylate adhesive injection
CEAP: Clinical Aetiology Anatomy Pathophysiology
CIVQ-14: Chronic Venous Insufficiency Questionnaire
CVD: Chronic venous disease
DUS: Duplex ultrasound
EQ5D: EuroQol’s 5-domain Utility Index
EQ-VAS: EuroQol’s Visual Analogue Scale
EVLA: Endovenous laser ablation
GSV: Great saphenous vein
MOCA: Mechanochemical ablation
NICE: The National Institute of Clinical Excellence
NTNT: Non-thermal non-tumescent
QoL: Quality of life
RFA: Radiofrequency ablation
SAE: Serious adverse events
SFJ: Saphenous-femoral junction
SPJ: Sapheno-popliteal Junction
SSV: Small saphenous vein
VAS: Visual Analogue Scale
VCSS: Venous Clinical Severity Score
